# Thrombospondin-1 (TSP-1) Stimulates Expression of Integrin *α*6 in Human Breast Carcinoma Cells: A Downstream Modulator of TSP-1-Induced Cellular Adhesion

**DOI:** 10.1155/2010/645376

**Published:** 2010-07-07

**Authors:** Anitha S. John, Vicki L. Rothman, George P. Tuszynski

**Affiliations:** ^1^Division of Pediatric Cardiology, Children's National Medical Center, George Washington University, Washington, DC 20052, USA; ^2^Department of Neuroscience, Center for Neurovirology, Temple University, 3500 North Broad Street, Philadelphia, PA 19140, USA

## Abstract

Thrombospondin-1 (TSP-1) is involved in a variety of different cellular processes including cell adhesion, tumor progression, and angiogenesis. This paper reports the novel finding that TSP-1 upregulates integrin *α*6 subunit in human keratinocytes and human breast cancer cells resulting in increased cell adhesion and tumor cell invasion. The effect of TSP-1 on *α*6 subunit expression was examined in human keratinocytes and breast adenocarcinoma cell lines (MDA-MB-231) treated with TSP-1 and in TSP-1 stably transfected breast cancer cells. TSP-1 upregulated *α*6 message and protein in these cells as revealed by differential display, Northern and Western blot analysis and immunohistochemical localization studies. The increased expression of *α*6 was shown to mediate adhesion and invasion of these cells to laminin, a major component of the basement membrane and extracellular matrix (ECM). These data suggest that TSP-1 plays an integral role in the attachment of cells to the ECM facilitating cell motility and angiogenesis.

## 1. Introduction

Adhesion to extracellular matrix (ECM) proteins is involved in almost every aspect of tumor cell metastasis including adhesion of circulating tumor cells in the vascular bed, invasion through the basement membrane, and growth of the new metastasis at a distant site [1]. In addition, contact with the ECM stimulates intracellular signaling, regulating cell attachment, migration, angiogenesis, and invasion [2–4]. 

Cellular adhesion is one of the major functions of thrombospondin-1(TSP-1), a component of the ECM. TSP-1 is a 450 kDa glycoprotein, originally thought to be a platelet *α*-granule specific protein [5]. Our laboratory was the first to show that TSP-1 functions both as a tumor cell and platelet adhesive protein [6]. There are four motifs in TSP-1 which have been recognized as adhesive domains: (1) the N-terminal heparin binding domain and its association with cell surface heparin proteoglycans, (2) the CSVTCG sequences within the type 1 repeats and its association with CD36 and the CSVTCG receptor, (3) the RGD sequence within the type 3 repeats and its association with the *α*_v_ *β*_3_ integrins, and (4) the RFYVVMWK and IRVVM sequences in the C-terminal domain [7]. A number of cell types can attach to TSP-1 including endothelial cells and myoblasts [8–10]. In addition, many tumor cell types such as osteosarcoma cells, melanoma cells, and breast cancer cells also adhere to TSP-1 [11–13]. This suggests that TSP-1 attachment can facilitate movement through the ECM.

There are several integrins that bind TSP-1. The best characterized interaction is with *α*_v_ *β*_3_ integrin and TSP-1, resulting in adhesion of a variety of cell types including platelets, melanoma cells, endothelial cells, and smooth muscle cells [14]. Other integrins that serve as TSP-1 receptors include *α*_IIb_ *β*_3_,  *α*_2_ *β*_1_,  *α*_3_ *β*_1_,  *α*_4_ *β*_1_,  *α*_9_ *β*_1_, and *α*_6_ *β*_1_ [15]. 

Integrins are a family of cell surface glycoproteins that function as receptors for ECM proteins, mediating both cell-substratum and cell-cell adhesion. Integrins are noncovalent, heterodimeric complexes of an *α* subunit and a *β* subunit [16]. To date there are 18*α* and 8*β* subunits which can associate to form at least 24 heterodimers [17, 18]. Because integrins play a major role in cell adhesion, it is not surprising that there is frequently altered expression and function of integrins in various tumor types [19]. Specifically, the *α*6 integrins have higher levels of expression in squamous carcinoma, small cell lung carcinoma, and bladder cancer [20]. Increased integrin *α*6*β*4 levels have also been associated with a decreased survival rate in patients with bladder cancer [21]. In addition, there has been evidence that integrin *α*6 expression is necessary for the tumor-like properties of a breast cancer stem cell like subpopulation [22].

Although the involvement of TSP-1 and integrins thus far are that of ligand and receptor, in this study, we report for the first time that TSP-1 is capable of upregulating *α* *6* expression in breast adenocarcinoma cells. Through differential display analysis, we determined TSP-1 stimulated increased *α* *6* mRNA levels in human keratinocytes. Given TSP-1 promotes metastasis and integrin *α*6 expression and function is abnormal in tumor cells, we then examined the effect of TSP-1 on integrin *α*6 expression in breast adenocarcinoma cell lines MDA-MB-231 and MDA-MB-435. Subsequent adhesion and invasion assays demonstrate a functional significance of this *α*6 expression with increased adhesion and increased tumor cell invasion. We hypothesize that TSP-1 not only acts as an adhesive protein itself, but also facilitates the adhesion of tumor cells to other extracellular matrix proteins, via upregulation of integrin *α*6 expression. Our results not only apply to breast cancer tumorigenesis but may have a direct bearing on TSP-1-mediated mechanisms of tumor angiogenesis. 

## 2. Materials and Methods

### 2.1. Materials

All reagents, unless specified otherwise, were reagent grade and purchased from Sigma Chemical Co. (St. Louis, MO). Tissue culture supplies were purchased from Fisher Scientific (Malvern, PA). Reagents for SDS-PAGE were purchased from Bio-Rad Laboratories (Richmond, CA). Laminin (type 1), type IV collagens and fibronectin were purchased from Collaborative Research (Bedford, MA). Rat monoclonal anti-integrin *α*6 (clone NK1-GoH3) and mouse monoclonal anti-integrin *α*6 (clone 1A10), prepared against a C-terminal peptide of the *α*6 heavy chain were purchased from Chemicon (Millipore/Chemicon, Billerica, MA). Goat polyclonal antihuman TSP-1 IgG and mouse monoclonal antihuman TSP-1 were made in our laboratory. 

### 2.2. Boyden Chamber Invasion Assay

Breast tumor cell invasion was measured using the modified Boyden chamber. Polycarbonate filters, 8 *μ*m pore size (Millicell, Millipore Corporation, Bedford, MA), were coated with 100 *μ*g type IV collagen (1 mg/mL 60% EtOH) and dried overnight at 25°C. Blind-well Boyden chambers were filled with 700 *μ*L of serum-free media containing 0.1% BSA in the lower compartment, and the coated filters were mounted in the chamber. Approximately 50,000 cells (tested to be greater that 95% viable) suspended in 300 *μ*L of the same media were placed in the upper chamber of the apparatus and allowed to settle onto the collagen-coated membrane. TSP-1 at a concentration of 133 nM (60 *μ*g/mL) was added in serum free media containing 1% albumin in the lower chamber and any neutralizing antibodies (10 *μ*g/mL IgG) as well as peptides were placed in the upper chamber. After an incubation period of 3–6 h at 37°C, the cells on the upper surface of the filter were removed with a cotton swab. The filters were fixed in 3% glutaraldehyde solution and stained with 0.5% crystal violet solution. Invasive cells adhering to the under-surface of the filter were counted using a phase contrast microscope (400X). The data were expressed as the summation of the number of invasive tumor cells in five representative fields.

### 2.3. Cell Culture and Treatment

The human breast adenocarcinoma cell line MDA-MB-231 was purchased from the American Type Culture Collection (CRL 10317, Rockville, MD). The TSP-1-transfected breast adenocarcinoma cell lines derived from MDA-MB-435 cells were obtained from Dr. David Roberts, NCI. These include three lines: a vector control (TH5), an intermediate TSP-1 producer (TH29), a high TSP-1 producer (TH26), and a carboxyl terminal truncated TSP-1 producer (TH50). The origin of the MDA-MB-435 cell line has been in question with some studies suggesting that the line was identical to a M14 melanoma line, however recent published data is consistent with both M14 and MDA-MB-435 cell lines being of breast cancer origin [23, 24]. Primary keratinocytes were obtained from Dr. Vicki Werth at the University of Pennsylvania and grown in keratinocyte media (Sigma Chemical Co). All cultures were kept in 5% CO_2_ at 37°C. Cells were cultured in 6-well plates for integrin *α*6 staining or T75 flasks for RNA isolation. Cells were grown to 85% confluence and were washed and incubated in serum-free medium containing 0.1% BSA. Different concentrations of TSP-1 and/or antibodies or peptides were added for a 1 to 72 h incubation. Cell viability after treatments was monitored by the trypan blue exclusion assay.

### 2.4. Cell Adhesion Assay

Wells of ninety-six well plates were coated with 0.1 mL of either 50 *μ*g/mL laminin, 50 *μ*g/mL collagen type IV, or 1% BSA for 60 minutes at 37°C. The wells were aspirated, treated with 200 *μ*L PBS containing 1% BSA for 1 hour and washed three more times with 200 *μ*L of PBS. Cells were incubated in TSP-1 or control buffer for 24 hours before being harvested. Cells were harvested and washed two times in serum-free DMEM and suspended in DMEM with the appropriate treatment at a final concentration of 5 × 10^4^ cells/100 *μ*L. Aliquots of 100 *μ*L were added to the wells and incubated at 37°C for 60 minutes or until cells have attached and spread. Nonadherent cells were removed by aspiration and the wells were washed three times with PBS. The total cell associated protein was determined directly by dissolving the attached cells in the microtiter wells with 200 *μ*L of the Pierce BCA working solution. The plate was covered with an adhesive mylar sheet and incubated at 60°C for 30 minutes. After cooling to room temperature and removing cover sheets, the absorbance of each well was measured at 562 nm with a microtiter plate reader (Biotek, Burlington, VT).

### 2.5. Differential Display

One one-base anchored oligodeoxy thymidylic acid primer HT11G (5′-AAGCTTTTTTTTTTTG-3′) was used to reverse transcribe mRNA from keratinocytes into first-strand cDNA, which was amplified subsequently by PCR using the arbitrary upstream primers H-AP5 (5′-AAGCTTAGTAGGC-3′) and H-AP8 (5′-AAGCTTTTACCGC-3′). Protocols were followed as described by Liang and Pardee [25]. PCR products were analyzed on a 6% DNA sequencing gel. The bands that showed either upregulation or downregulation were cut out from the gel, eluted, and amplified by PCR.

### 2.6. Cloning and Sequencing

The reamplified cDNA bands were cloned using the TA cloning kit (Invitrogen, SanDiego, CA). The isolated fragment was sequenced using an automated DNA sequencer (Core Sequencing Facility, Drexel School of Medicine). The sequence was then analyzed through an NCBI Blast search.

### 2.7. Immunohistochemical Staining

The rat antihuman integrin *α*6 antibody, GoH3, was used for cell staining. Rat IgG controls were used. TSP-1-transfected cell lines and MBA-MD-231 cells treated with exogenous TSP-1 were grown to 85% confluence on glass slides. The cells were then fixed with 2.5% glutaraldehyde and stained with the avidin-biotin immunoperoxidase complex technique (Vectastain Elite ABC Kit, Vector Laboratories). 

### 2.8. Northern Blot Analysis

Total RNA was isolated from tumor cells by Rneasy Total RNA Kits (QIAGEN Inc. Chatsworth, CA) following the manufacturer's direction. 15 *μ*g of total RNA was subjected to electrophoresis on 1% agarose/formaldehyde gels and blotted onto nylon paper. The paper was hybridized with the appropriate ^32^P-labeled cDNA probes and autoradiographed at −80°C with an intensifying screen. After recording the results, the same paper was re-probed with a beta-actin cDNA probe and exposed at −80°C. The cDNAs were radiolabeled with ^32^P by the random primer labeling method using the Stratagene labeling kit (Stratagene, La Jolla, CA).

### 2.9. Thrombospondin-1 Purification

TSP-1 was purified from Ca^+2^ ionophore A23187-activated platelets in our laboratory as previously described [26]. Purity was assessed by SDS-PAGE using Coomassie blue or silver staining. All TSP-1 used was further purified to remove all bound TGF-*β*1 according to the procedure of Murphy-Ullrich et al. [27]. TGF-*β*1 levels were monitored by human TGF-*β*1 ELISA kits (Quantikine, R&D Systems, Minneapolis, MN).

### 2.10. Western Blot Analysis

Total SDS-detergent lysate of breast cancer cells was fractionated on a 10% SDS-PAGE and then transferred to a PVDF membrane using a Pharmacia Phast gel electrophoresis system. Nonspecific sites of the membranes were blocked with 5% skim milk in Tris-buffered saline containing 0.1% Tween 20 (TBS-T) for 1 h. The immunoblots were incubated with primary antibodies diluted in TBS-T for 1 h at a concentration of 1 *μ*g/mL. After washing, the immunoblots were incubated in TBS-T with the appropriate horseradish peroxidase-conjugated secondary antibodies for 1 h at a concentration of 0.1 *μ*g/mL. The immunoreactivity was detected using enhanced chemoluminesence (ECL) system (Amersham, Arlington Heights, IL).

## 3. Results

### 3.1. Identification of *α* *6* Upregulation

**Figure 1:**
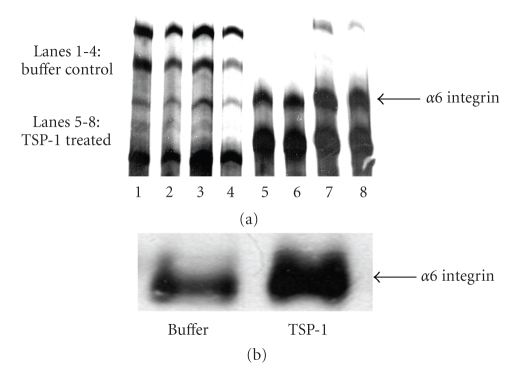
TSP-1 upregulates integrin *α* *6* subunit message in human keratinocytes. Keratinocytes were treated with either bis-tris-propane buffer or TSP-1 (60 *μ*g/mL) in serum-free media and mRNA expression was analyzed by (a) differential display analysis and confirmed with (b) northern blot analysis. Experiments were repeated three times and the results of a representative experiment are shown in the figure.

Differential display analysis was performed on keratinocytes treated with either 20 mM bis-tris propane buffer (vehicle for TSP-1) or TSP-1 (60 *μ*g/mL) for twenty-four hours. The mRNA from these cells was collected and used for differential display RT-PCR. Several bands were found to be upregulated (Figure 1(a)) between buffer and TSP-1-treated samples in two separate mRNA samples collected from independent experiments. The upregulation of one band was confirmed using Northern blot analysis (Figure 1(b)). The isolated band (236 bp) was cloned and sequenced and was found to show 98% homology to the integrin *α*_6_ subunit. 

### 3.2. The Effect of TSP-1 on Integrin *α*6 mRNA Production in Human Breast Cancer Cells

**Figure 2:**
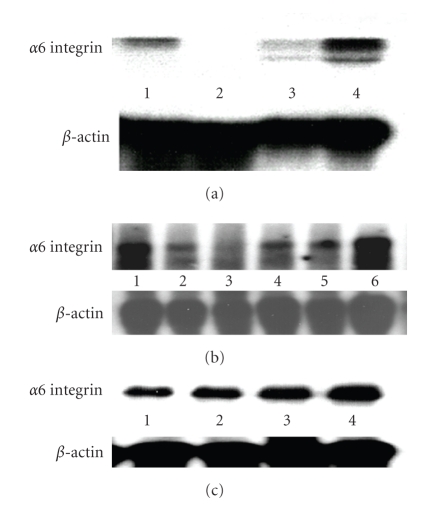
TSP-1 induces integrin *α* *6* mRNA expression in human breast cancer cells. Two breast cancer cell lines (a) stably transfected TSP- 1 MDA-MB-435 cell lines with variable TSP-1 expression and (b) MDA-MB-231 cells treated with either buffer or TSP-1 (60 *μ*g/mL) were analyzed by northern blot analysis for integrin *α*6 expression. TSP-1 dose dependent response of integrin *α*6 was then assessed in the MDA-MB-231 (c). Blots were normalized to a 2.4 *β*-actin probe. A pancreatic carcinoma cell line, BxPC3, was used as a positive control. (a) TSP-1 stably transfected TSP-1 cells. 1: BxPC3 cell line, 2: TH5 cells (vector control), 3: TH29 cells (intermediate TSP-1 producer), and 4: TH26 cells (high TSP-1 producer). (b) MDA-MB-231 cells. 1: BxPC3 cell line, 2: buffer treatment, 3: TSP-1 (1 hour), 4: TSP-1 (6 hours), 5: TSP-1 (12 hours), and 6: TSP-1 (24 hours). Experiments were repeated three times and the results of a representative experiment are shown in the figure. (c) MDA-MB-231 cells. 1: Buffer treatment, 2: TSP-1 (20 *μ*g/mL), 3: TSP-1 (40 *μ*g/mL), and 4: TSP-1 (60 *μ*g/mL).

To examine if TSP-1 upregulates integrin *α* *6* production in human breast cancer cells, MDA-MB-231 cells were treated with TSP-1 (60 *μ*g/mL) and total RNA was collected at various time points including 1 hour, 6 hours, 12 hours, and 24 hours. The 236 base pair band isolated through differential display was used as the probe for northern blot analysis. The results show that integrin *α* *6* expression appears to increase after a 24 hour incubation with TSP-1 with some downregulation seen at the one hour time point (see Figure 2(b)). In addition, TSP-1 stably transfected MDA-MB-435 cells were also examined for integrin *α* *6* mRNA expression. Three cells lines were examined: a vector control (TH5), an intermediate TSP-1 producer (TH29), and a high TSP-1 producer (TH26). The results show that the highest level of integrin *α*6 mRNA occurred in the high TSP-1 producer (TH26) (Figure 2(a)). The intermediate producer showed more integrin *α*6 mRNA production than the vector control (TH5) but less than the TH26 cell line. MDA-MB-231 cells were then incubated with varying concentrations of TSP-1 for a period of 24 hours. The highest level of expression occurred with a TSP-1 dosage of 60 *μ*g/mL. This data support the conclusion that TSP-1 not only upregulates integrin *α*6 mRNA expression, but that it does so in a dose and time dependent fashion.

### 3.3. Expression of Integrin *α* *6* Protein in Human Breast Carcinoma Cell Lines

**Figure 3:**
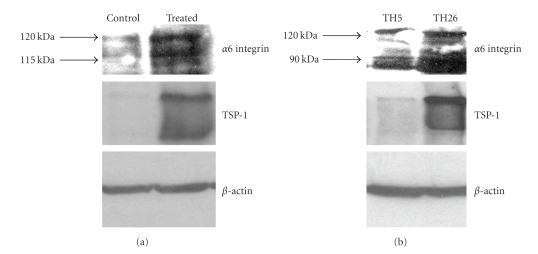
TSP-1 induces integrin *α* *6* protein expression in human breast cancer cells. MD-MBA-231 cells (a) and MD-MBA-435 cells (b) were grown in six well tissue culture plates in serum-free media either with or without 60 *μ*g/mL TSP-1 for 24 hours. Cell extracts were prepared with SDS-sample buffer, reduced with 5%  *β*-mercaptoethanol, and separated on 10% SDS-PAGE. Blots were probed with 1 *μ*g/mL of mouse *α*6 integrin IgG and followed by 0.1 *μ*g/mL HRP-coupled rabbit antimouse IgG and developed using enhanced chemoluminesence. Experiments were repeated two times and the results of a representative experiment are shown in the figure.

MDA-MB-231 cells were treated with either buffer or TSP-1 (60 *μ*g/mL) for 24 hours and the cells were lysed with SDS-PAGE sample buffer and analyzed for *α*6 protein expression by Western blot analysis. The TSP-1-treated cells expressed a significant increase in a 120 kilodalton band consistent with the heavy chain of the *α*6 subunit (Figure 3(a)). A slightly smaller band of approximately 115 kilodaltons was also seen in the blot consistent with the presence of a splice variant or degradation product of the *α*6 subunit. Similarly, when the blot of the whole cell lysate of the TSP-1 stably transfected breast cell line, TH26, was compared to the vector control TH5, we saw a marked expression of 90 and 120 kilodalton bands when the blots were probed with an antibody specific for the heavy chain of the *α*6 subunit (Figure 3(b)). The blots were also probed with an antibody to TSP-1 confirming that either TSP-1 was added exogenously or that the cell expressed their own TSP-1 (second panel in Figures 3(a) and 3(b)). Equal loading of the samples was confirmed by probing the extracts with an antibody to *β*-actin. These results confirm the Northern Blot results indicating that TSP-1 not only upregulates integrin message but also protein.

**Figure 4:**
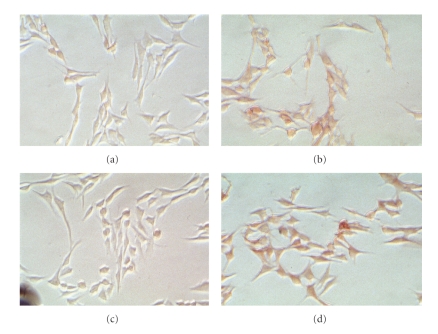
TSP-1 stably transfected cells express integrin *α*6. Cells were grown in six well chamber slides, fixed, and stained with rat *α*6 integrin IgG as described in Section 2. Cells were photographed at 200X magnification. (a) TH5 cells (vector control). (b) TH29 cells, (c) TH50, (d) TH26 cells. Experiments were repeated three times and the results of a representative experiment are shown in the figure.

To further show that *α*6 expression is dependent on TSP-1 expression, the panel of TSP-1 expressing cells were fixed with 2.5% gluteraldehyde and immunohistochemically stained with a rat monoclonal antibody specific against the heavy chain of *α*6 (Figure 4). When the TSP-1 transfected cell lines TH29, TH26 and TH50 were examined, only TH29, and TH26 showed significant expression of *α*6, while the vector control line TH5 and TH50 cell line, expressing a TSP-1 mutant missing the carboxyl terminus were negative. These results show that *α*6 expression is dependent on expression of full-length TSP-1. 

### 3.4. Establishing Specificity of TSP-1-Induced Integrin *α* *6* Expression

**Figure 5:**
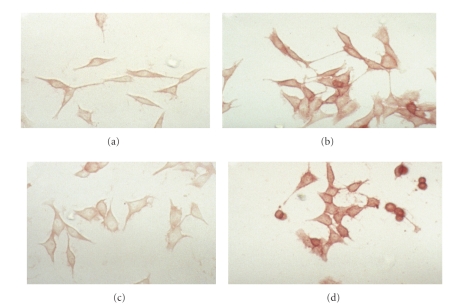
Anti-TSP-1 antibody inhibition of integrin *α*6 production in TSP-1 stably transfected cells. Cells were grown in six well chamber slides in either serum-free media or media containing either 10 *μ*g/mL control IgG or 10 *μ*g/mL goat antihuman TSP-1 IgG, fixed, and stained with rat *α*6 integrin IgG as described in Section 2. Cells were photographed at 200X magnification. (a) TH5 cells (vector control). (b) TH26 cells (high TSP-1 producer). (c) TH26 cells plus anti-TSP-1 antibody. (d) TH26 cells plus control IgG.

To determine if the staining observed in the stably transfected cells was specific to TSP-1, the TH26 cells were treated with either 10 *μ*g/mL of polyclonal goat anti-TSP-1 IgG or 10 *μ*g/mL of goat IgG and grown for an additional 24 hours. Immunohistochemical staining showed decreased expression of integrin *α*6 in the TH26 cells treated with the anti-TSP-I IgG comparable to the TH5 vector control, while the control antibody IgG-treated cells and untreated cells showed significant *α*6 staining (Figure 5). When the same experiment was repeated using an antitype 1 repeat TSP-1 antibody, there was no blocking effect (data not shown). These results suggest that endogenously produced TSP-1 specifically induces integrin *α* *6* upregulation through domains other than the type 1 repeat domain of TSP-1.

### 3.5. The Functional Significance of TSP-1-Induced Integrin *α* *6* Expression on Tumor Cell Adhesion

**Figure 6:**
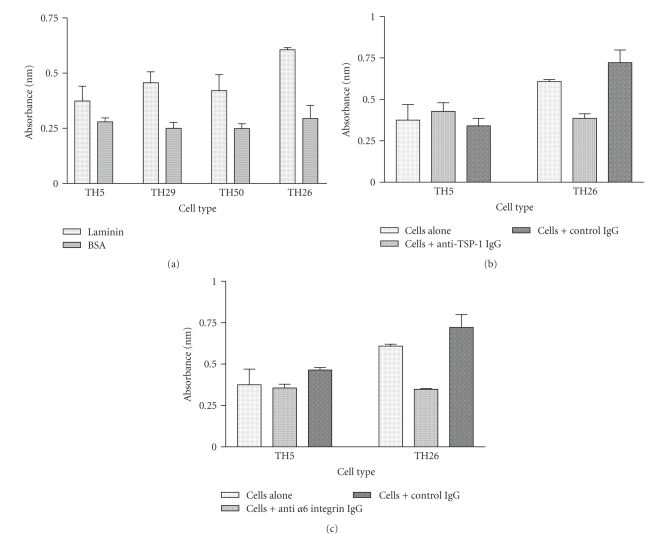
High endogenous TSP-1 production increases cell adhesion to laminin. Stably transfected MDA-MB-435 cells were either incubated alone (a), with either 10 *μ*g/mL control IgG or 10 *μ*g/mL goat antihuman TSP-1 IgG (b), or either 10 *μ*g/mL control IgG or 10 *μ*g/mL rat antihuman *α*6 integrin IgG (c), and assessed for adhesion to laminin as described in Section 2. BSA was used as a negative control. The error bars represent the standard error of the mean of triplicate samples and the experiment was repeated three times with similar results.

TSP-1 stably transfected breast cancer cell lines were tested for adhesion to laminin, the major adhesive ligand of *α*6*β*1 (Figure 6). We found that only the high TSP-1 expressing cell line TH26 showed a significantly higher (>50%) extent of adhesion to laminin (*P* < .05), when compared to either the vector control line TH5 or TH50, line expressing the carboxyl domain-truncated TSP-1 (Figure 6(a)). The TH29 cell line also had a significantly higher level of adhesion to laminin as compared to BSA (*P* < .05), but the results were not as impressive as the high TSP producing cell line (TH26). Adhesion of TH5 and TH50 cell lines to laminin were statistically the same and statistically indistinguishable from adhesion to bovine serum albumin (BSA), the negative control (Figure 6(a)). 

To show that the adhesion of TH26 cell line to laminin was TSP-1 and *α*6-integrin dependent, blocking experiments with either a polyclonal TSP-1 antibody or an anti-integrin *α* *6* monoclonal were performed (Figures 6(b) and 6(c)). When TH26 cells were preincubated for 24 hours with either 10 *μ*g/mL of polyclonal goat anti-TSP-1 IgG or 10 *μ*g/mL of rat monoclonal anti-integrin *α*6 (clone NK1-GoH3) IgG, adhesion of the cells was reduced to levels observed with the vector control line THP5 (Figures 6(b) and 6(c)). In contrast the respective control IgGs had no effect on adhesion and the extent of adhesion was statistically indistinguishable from untreated cells (compare bar labeled cells alone with bar labeled cell plus IgG, *P* > .5, in Figures 6(b) and 6(c), resp.). These results strongly suggest that that adhesion of TH26 cells to laminin is dependent on both TSP-1 and integrin *α*6.

### 3.6. The Effect of TSP-1-Induced Integrin *α*6 Expression in Tumor Cell Invasion through Laminin

**Figure 7:**
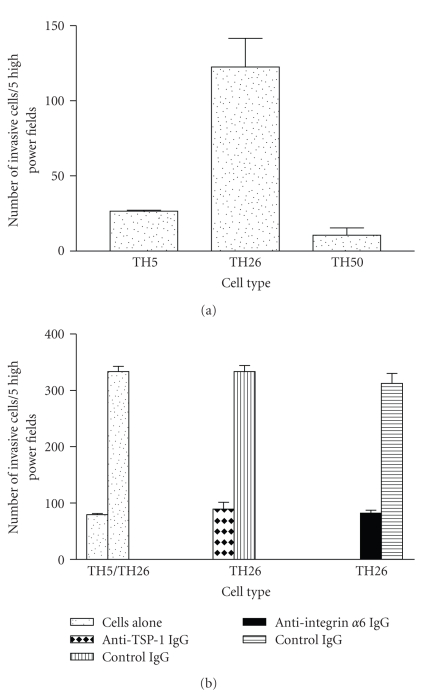
High endogenous TSP-1 production increases cell invasion of laminin. Tumor cell invasion of laminin was performed as described in Section 2. Invasion of cells untreated (a), invasion of cells pretreated with either with either 10 *μ*g/mL control IgG or 10 *μ*g/mL goat antihuman TSP-1 IgG or 10 *μ*g/mL rat antihuman *α*6 integrin IgG (b). The error bars represent the standard error of the mean of triplicate samples and the experiment was repeated three times with similar results.

Tumor cell invasion of laminin was dependent on TSP-1 and *α*6*β*1 expression (Figure 7). After five hours of incubation, the high TSP-1 producer cell line TH26 was 5-fold more invasive than either the TH5 vector controls or TH50 cells expressing carboxyl truncated TSP-1 (Figure 7(a)). Using a six-hour incubation period, the experiment was repeated in the presence of either 10 *μ*g/mL of polyclonal goat anti-TSP-1 IgG or 10 *μ*g/mL of rat monoclonal anti-integrin *α*6 (clone NK1-GoH3) IgG (Figure 7(b)). At baseline, again the TH26 cells showed 5-fold more invasion than the TH5 cells. Incubating the TH26 cells with either a polyclonal TSP-1 antibody or a neutralizing integrin *α*6 antibody completely reduced invasion to the level of the TH5 cells. Taken together, these data provide strong evidence that tumor cell adhesion and migration are influenced by TSP-1-mediated integrin *α*6 expression and are consistent with our previous studies showing that TSP-1 promotes the invasion of breast cancer cells [13].

## 4. Discussion

TSP-1 expression has been examined quite extensively in a variety of tumor types. TSP-1 localizes strongly in the desmoplastic stroma surrounding the tumor in head and neck cancers, pancreatic cancer, and breast carcinoma [28]. In addition, a variety of tumor cells are capable of secreting TSP-1 including pancreatic tumor cells, squamous lung carcinoma, and breast cancer cells [7]. TSP-1 is involved in both tumor cell adhesion and tumor cell invasion through multiple mechanisms including several adhesive domains within TSP-1 itself and upregulation of enzymes such as matrix metalloproteinase 9 (MMP-9) [29, 30].

This report describes the novel observation that TSP-1 stimulates integrin *α* *6* expression in human breast carcinoma cells. Although TSP-1 and integrins have been studied, the relationship thus far described has been that of receptor and ligand. We have discovered through differential display that TSP-1 is able to upregulate integrin *α* *6* message in human keratinocytes. When we subsequently examined two human breast cancer lines, the MDA-MB-231 cell line and a TSP-1 stably transfected MDA-MB-435 cell line, integrin *α* *6* mRNA expression increases with both exogenous treatment and endogenous expression of TSP-1. Western blot analysis with anti-*α*6 antibody of breast cancer cells stably transfected with TSP-1 or cells treated with TSP-1 revealed the upregulation of bands of 80–120 kDa consistent with the heavy chain of *α*6 subunit. In addition, immunohistochemical analysis of the TSP-1 stably transfected cells showed that the high TSP-1 producers (TH26) were positive for integrin *α*6 protein expression as compared to the vector control (TH5), the intermediate TSP-1 producer (TH29), or the carboxyl terminally truncated TSP-1 producer (TH50). Repeating the staining after treating with anti-TSP-1 polyclonal antibodies resulted in markedly decreased expression of integrin *α*6 protein to the level of the TH5 cells. As stated above, TSP-1 is not only produced by tumor cells but also by cells within the tumor stroma, such as fibroblasts. This upregulation of integrin *α*6 by TSP-1, both exogenous and endogenous, suggests that TSP-1 contributes to tumor cell adhesion by both direct production by tumor cells and by exposure to exogenous TSP-1 within the desmoplastic stroma. Potential therapeutics will need to target not only TSP-1 within the extracellular matrix, but also that produced by the tumor cell itself. These results indicate that TSP-1 upregulates integrin *α*6 protein both at the message and protein level and is one of the matrix proteins involved in tumor cell regulation of cell surface receptors needed for tumor progression. 

The functional significance of TSP-1's effect on stimulating integrin *α*6 production was examined through both cell adhesion and cell invasion assays. When examining the stably transfected cells in a cell adhesion assay, the TH26 cells showed a significantly higher level of adhesion than the TH5 or TH50 cell lines. The intermediate producers of TSP-1, the TH29 cells, did show an increase in adhesion to laminin, but not to the same extent as the TH26 cell line. This reflects the dose-dependent effect that TSP-1 has on integrin *α*6 mRNA production. This adhesion to laminin was inhibited by a polyclonal anti-TSP-1 antibody and also by a monoclonal anti-integrin *α* *6* antibody showing that both proteins play a role in breast cancer cell adhesion to laminin, a major component of the basement membrane. Inhibiting cell adhesion with both antibodies also shows that the cell adhesion observed was not only due to TSP-1's known ability as a promoter of cell adhesion. Certainly, cell adhesion is a necessary feature of tumor progression and metastasis. In order for a tumor cell metastasis to occur, the cell must initially be able to attach to the extracellular matrix which then serves as a scaffold for the tumor to migrate and eventually invade.

The ability of the TSP-1-transfected breast cancer cells to invade was also assessed by an *in vitro* invasion assay. Invasion was markedly increased in the TH26 cells when compared to the TH5 cells and the TH50 cells. Neutralizing antibodies against either TSP-1 or integrin *α*6 were able to significantly reduce invasion through the laminin-coated filter. The capacity of the TH26 cells to invade at such a high rate implies that the expression of the *α* *6* integrins does not inhibit motility of these cells. In fact, it appears to enhance motility and invasion likely due to the TSP-1's known effect on upregulating proteolytic systems, such as the matrix metalloproteinase system and the urokinase system [31, 32]. This, in combination with increased integrin *α*6 production and TSP-1's known ability to serve as a cell adhesive protein, facilitates both the attachment of tumor cells to the ECM and the migration required for tumor progression.

TSP-1 levels in serum of patients have been shown to be higher in patients with colorectal cancer with venous invasion as compared to patients without venous invasion [33]. A similar study with gynecological malignancies also showed higher serum levels in patients with cancer and that TSP-1 levels increased with higher grades of malignancy [34]. As seen with the cell adhesion assay, TSP-1 can have a varying effect depending on the amount produced by the tumor cell which could account for the more metastatic phenotype seen in those patients with higher levels of TSP-1. Using serum levels may also be useful as a biomarker for metastatic potential. One important aspect of this study not examined in depth here is the *β* subunit association with *α*6. The *α*6 subunit is capable of complexing to either the *β*1 or the *β*4 subunit although it has been reported to bind the *β*4 subunit preferentially [16]. Initial immunoprecipitation experiments done in our laboratory point to the colocalization of the *α*6 subunit with the *β*4 subunit. This data needs to be further substantiated and is the subject of another study.

The increase in tumor cell invasion seen in the invasion assay is in part due to TSP-1 upregulation of integrin *α*6 protein and facilitation of adhesion, but is also due to the downstream proteolytic systems that are activated by TSP-1. TSP-1 has been shown to upregulate TGF-*β* production which has been shown to be one of the mechanisms for TSP-1-induced matrix metalloproteinase-9 and urokinase production [27]. These proteolytic enzymes have been shown to be involved in TSP-1-induced tumor cell invasion [28, 30]. The effects of TSP-1 on cell adhesion through integrin *α*6 act in complement with the increased proteolytic enzymes further facilitating metastasis.

TSP-1 is a complex molecule with multiple mechanisms of action [29]. These different mechanisms are thought to be mediated by the many different domains within the 150 kDa TSP-1 molecule. The type 1 repeats in TSP-1 have been shown to be involved in the regulation of downstream proteolytic enzymes such as MMP-9 and urokinase, but in our results, an anti-TSP-1 type 1 repeat antibody was unable to inhibit expression of TSP-1-induced integrin *α*6 production as assessed by immunohistochemical staining. This implies that the type 1 repeats are not responsible for integrin *α*6 upregulation, but are important in tumor cell invasion through stimulating expression of matrix degrading enzymes. The multiple domains of TSP-1 account for the multiple mechanisms of action contributing to tumor cell metastasis, including adhesion to the various components of the extracellular matrix which is a critical feature of further metastasis. 

In summary, our data show that TSP-1 upregulates integrin *α*6 subunit expression both at the message and protein level in breast cancer cells. This upregulation promotes tumor cell adhesion to laminin, and subsequently aids in tumor cell invasion. The novel observation that endogenous TSP-1 is capable of stimulating expression and possible activation of the *α* *6* integrins warrants further study especially concerning the mechanisms involved in tumor progression. The roles of integrin *α*6 in TSP-1-mediated effects such as angiogenesis and its interaction with other TSP-1-mediated proteins are still yet to be determined and provide an exciting area of new research.

## References

[1] Albo D, Berger DH, Wang TN, Hu X, Rothman V, Tuszynski GP (1997). Thrombospondin-1 and transforming growth factor-beta1 promote breast tumor cell invasion through up-regulation of the plasminogen/plasmin system. *Surgery*.

[2] Heino J (1996). Biology of tumor cell invasion: interplay of cell adhesion and matrix degradation. *International Journal of Cancer*.

[3] Song S-Y, Nomizu M, Yamada Y, Kleinman HK (1997). Liver metastasis formation by laminin-1 peptide (LQVQLSIR)-adhesion selected B16-F10 melanoma cells. *International Journal of Cancer*.

[4] Haas TL, Davis SJ, Madri JA (1998). Three-dimensional type I collagen lattices induce coordinate expression of matrix metalloproteinases MT1-MMP and MMP-2 in microvascular endothelial cells. *The Journal of Biological Chemistry*.

[5] Lawler JW, Slayter HS, Coligan JE (1978). Isolation and characterization of a high molecular weight glycoprotein from human blood platelets. *The Journal of Biological Chemistry*.

[6] Tuszynski GP, Gasic TB, Rothman VL, Knudsen KA, Gasic GJ (1987). Thrombospondin, a potentiator of tumor cell metastasis. *Cancer Research*.

[7] Qian X, Tuszynski GP (1996). Expression of thrombospondin-1 in cancer: a role in tumor progression. *Proceedings of the Society for Experimental Biology and Medicine*.

[8] Murphy-Ullrich JE, Hook M (1989). Thrombospondin modulates focal adhesions in endothelial cells. *Journal of Cell Biology*.

[9] Adams JC (1995). Formation of stable microspikes containing actin and the 55 kDa actin bundling protein, fascin, is a consequence of cell adhesion to thrombospondin-1: implications for the anti-adhesive activities of thrombospondin-1. *Journal of Cell Science*.

[10] McClenic BK, Mitra RS, Riser BL, Nickoloff BJ, Dixit VM, Varani J (1989). Production and utilization of extracellular matrix components by human melanocytes. *Experimental Cell Research*.

[11] Decker S, van Valen F, Vischer P (2002). Adhesion of osteosarcoma cells to the 70-kDa core region of thrombospondin-1 is mediated by the *α*4*β*1 integrin. *Biochemical and Biophysical Research Communications*.

[12] Ginsburg V, Roberts DD (1988). Glycoconjugates and cell adhesion: the adhesive proteins laminin, thrombospondin and bon Willebrand’s factor bind specifically to sulfated glycolipids. *Biochimie*.

[13] Wang TN, Qian X-H, Granick MS (1996). Thrombospondin-1 (TSP-1) promotes the invasive properties of human breast cancer. *Journal of Surgical Research*.

[14] Adams JC, Lawler J (1993). Diverse mechanisms for cell attachment to platelet thrombospondin. *Journal of Cell Science*.

[15] Staniszewska I, Zaveri S, Del Valle L (2007). Interaction of *α*9*β*1 integrin with thrombospondin-1 promotes angiogenesis. *Circulation Research*.

[16] Huveneers S, Truong H, Danen EHJ (2007). Integrins: signaling, disease, and therapy. *International Journal of Radiation Biology*.

[17] van der Flier A, Sonnenberg A (2001). Function and interactions of integrins. *Cell and Tissue Research*.

[18] Francis SE, Goh KL, Hodivala-Dilke K (2002). Central roles of *α*5*β*1 integrin and fibronectin in vascular development in mouse embryos and embryoid bodies. *Arteriosclerosis, Thrombosis, and Vascular Biology*.

[19] Chung J, Kim TH (2008). Integrin-dependent translational control: implication in cancer progression. *Microscopy Research and Technique*.

[20] Ohara T, Kawashiri S, Tanaka A (2009). Integrin expression levels correlate with invasion, metastasis and prognosis of oral squamous cell carcinoma. *Pathology and Oncology Research*.

[21] Grossman HB, Lee C, Bromberg J, Liebert M (2000). Expression of the *α*6ß4 integrin provides prognostic information in bladder cancer. *Oncology Reports*.

[22] Cariati M, Naderi A, Brown JP (2008). Alpha-6 integrin is necessary for the tumourigenicity of a stem cell-like subpopulation within the MCF7 breast cancer cell line. *International Journal of Cancer*.

[23] Chambers AF (2009). MDA-MB-435 and M14 cell lines: identical but not M14 melanoma?. *Cancer Research*.

[24] Hollestelle A, Schutte M (2009). Comment Re: MDA-MB-435 and M14 cell lines: identical but not M14 melanoma?. *Cancer Research*.

[25] Liang P, Pardee AB (1992). Differential display of eukaryotic messenger RNA by means of the polymerase chain reaction. *The Science*.

[26] Switalska HI, Niewiarowski S, Tuszynski GP (1985). Radioimmunoassay of human platelet thrombospondin: different patterns of thrombospondin and *β*-thromboglobulin antigen secretion and clearance from the circulation. *Journal of Laboratory and Clinical Medicine*.

[27] Murphy-Ullrich JE, Schultz-Cherry S, Hook M (1992). Transforming growth factor-*β* complexes with thrombospondin. *Molecular Biology of the Cell*.

[28] Albo D, Arnoletti JP, Castiglioni A (1994). Thrombospondin (TSP) and transforming growth factor beta 1 (TGF-*β*) promote human A549 lung carcinoma cell plasminogen activator inhibitor type 1 (PAI-1) production and stimulate tumor cell attachment in vitro. *Biochemical and Biophysical Research Communications*.

[29] Sargiannidou I, Qiu C, Tuszynski GP (2004). Mechanisms of thrombospondin-1-mediated metastasis and angiogenesis. *Seminars in Thrombosis and Hemostasis*.

[30] Qian X, Rothman VL, Nicosia RF, Tuszynski GP (2001). Expression of thrombospondin-1 in human pancreatic adenocarcinomas: role in matrix metalloproteinase-9 production. *Pathology and Oncology Research*.

[31] Albo D, Tuszynski GP (2004). Thrombospondin-1 up-regulates tumor cell invasion through the urokinase plasminogen activator receptor in head and neck cancer cells. *Journal of Surgical Research*.

[32] Chen J-Z, Wang S, Tang R (2002). Cloning and identification of a cDNA that encodes a novel human protein with thrombospondin type I repeat domain, hPWTSR. *Molecular Biology Reports*.

[33] Yamashita Y, Kurohiji T, Tuszynski GP, Sakai T, Shirakusa T (1998). Plasma thrombospondin levels in patients with colorectal carcinoma. *Cancer*.

[34] Nathan FE, Hernandez E, Dunton CJ (1994). Plasma thrombospondin levels in patients with gynecologic malignancies. *Cancer*.

